# Early Sucrose Degradation and the Dominant Sucrose Cleavage Pattern Influence *Lycoris sprengeri* Bulblet Regeneration In Vitro

**DOI:** 10.3390/ijms222111890

**Published:** 2021-11-02

**Authors:** Ziming Ren, Yunchen Xu, Xuesi Lvy, Dong Zhang, Cong Gao, Yefan Lin, Yue Liu, Yun Wu, Yiping Xia

**Affiliations:** 1 Genomics and Genetic Engineering Laboratory of Ornamental Plants, Department of Horticulture, College of Agriculture and Biotechnology, Zhejiang University, Hangzhou 310058, China; zimingren@zju.edu.cn (Z.R.); xuyunchen1998@icloud.com (Y.X.); 18667363280@163.com (X.L.); zhangdong8732@126.com (D.Z.); conggao@zju.edu.cn (C.G.); 21916198@zju.edu.cn (Y.L.); liuyuezju@163.com (Y.L.); 2 Department of Landscape Architecture, School of Civil Engineering and Architecture, Zhejiang Sci-Tech University, Hangzhou 310018, China

**Keywords:** 6-benzyladenine, bulblet regeneration, sucrose cleavage, endogenous hormone, cell wall invertase, *Lycoris* *sprengeri*

## Abstract

Bulblet formation and development determine the quantitative and qualitative traits, respectively, of bulb yield for most flowering bulbs. For *Lycoris* species, however, the underlying molecular mechanism remains elusive. Here, clonal bulblets of *Lycoris sprengeri* (*Ls*) derived from the same probulb were used as explants to establish efficient and inefficient in vitro regeneration systems by adjusting the 6-benzyladenine (BA) concentrations in media. BA application did not change the biological processes among groups but led to earlier decreases in sucrose and total soluble sugar (TSS) contents. Correlation analyses showed that the BA treatments changed the interaction between carbohydrate and endogenous hormone contents during bulblet regeneration. We found that two sucrose degradation enzyme-related genes, cell wall invertase (CWIN) and sucrose synthase, exhibited exactly opposite expression patterns during the competence stage. In addition, the regeneration system that obtained more bulblets showed significantly higher expression of *LsCWIN2* than those that obtained fewer bulblets. Our data demonstrate the essential role of BA in accelerating sucrose degradation and the selection of a dominant sucrose cleavage pattern at the competence stage of in vitro bulblet regeneration. We propose that a relatively active CWIN-catalyzed pathway at the competence stage might promote bulblet regeneration, thus influencing bulb yield.

## 1. Introduction

*Lycoris* is a monocotyledonous genus belonging to the Amaryllidaceae family and having high medicinal, ornamental and ecological value [1,2,3,4,5]. *Lycoris sprengeri* (*Ls*) is an endemic *Lycoris* species in China [6] that has become an important ground cover for urban landscaping in recent years [7]. This plant features vibrant, blue-purple flowers with strong resistance and low maintenance costs. However, limited outgrowth of bulblets (axillary buds) and an extended juvenile period have led to low reproductive efficiency of *Ls* under natural conditions [4]. The increasing demand for *Ls* bulbs has already resulted in significant resource diminishment and heavy exploitation of their natural habitats. To address this situation, our previous research developed an effective in vitro bulblet multiplication protocol [4], thus providing a series of clonal bulblets derived from one individual seed, as native *Lycoris* from different genetic backgrounds is not suitable for current research.

Tissue culture has the potential to build commercial stocks of ornamental geophytes with desired characteristics [8], and has been used for the production of flower bulbs with long juvenile phases and high genetic variability [7,9,10]. For the mass multiplication of desirable cultivars and conservation of endangered species, in vitro propagation of *Lycoris* species has been developed and optimized since the 1980s. The predominant in vitro propagation method for *Lycoris* explants is via a direct route by the propagation of the already existing axillary meristems (AxMs) or the formation of adventitious meristems (AdMs). Thus, the formation of AxMs and/or AdMs and the subsequent development of propagules determine the quantitative (number) and qualitative (volume) traits of bulb yield, respectively. A limited number of lateral meristems is the main factor limiting the regeneration efficiency of flowering bulbs [8]. To date, many studies have focused on the optimization of culture conditions for bulblet formation (induction), development (bulbing) and nutrient accumulation in vitro. However, the molecular mechanisms involved in bulblet formation in vitro and the effect of culture conditions on the proliferation rate are still poorly understood.

Bulblet formation and development are regulated by several factors, including carbohydrate metabolism and endogenous hormone regulation [11,12,13]. In *Lycoris* species, bulblets are derived from scale axils, during which starch granules available for degradation in scales serve as an important energy source and allow for scales to be sink organs [4]. As the main storage compounds in *Lycoris* scales, starch was shown to continuously decrease during bulblet initiation and development in *Lycoris radiata* via cross-cutting ex vitro, during which the starch content in the outer scales (OSs) decreased at a relatively faster rate than it did in the inner scales (ISs) [14]. Soluble sugars degraded from starch provide carbon and energy for plant morphogenesis [11]. Increasing content of soluble sugars in ISs of *L. radiata* promotes the expression levels of *CycD* (*D-type cyclin*) genes and accelerates cell division during bulb development [13]. Stage-specific upregulation of genes encoding sucrose metabolism (sucrose synthase, SuSy), UDP-glucose pyrophosphorylase (UGPase), and starch synthesis enzymes (starch synthase (SS), granule-bound starch synthase (GBSS), ADP glucose pyrophosphorylase (AGPase)) suggests their important roles in bulblet initiation and development, respectively [14]. To date, studies on the changes in carbohydrate contents and the expression of related genes during bulblet formation and development in flowering bulbs have revealed strong regulation of sucrose and starch metabolism during this process [11,13,14,15,16,17]. However, changes in early carbohydrate metabolism and their relationship with bulblet formation capability during in vitro bulblet regeneration of *Lycoris* remain unclear.

The fine-tuned interactions between nutrients and hormones play pivotal roles in orchestrating plant yield and resistance to abiotic and biotic constraints [18,19]. Sugars and cytokinins (CKs) are among the most central regulators during plant growth and development. Briefly, sugars serve as structural components, energy sources and signaling entities throughout plant life [18,20,21,22]. In autotrophic organisms, most carbohydrates make up the bulk of the biomass, and the crop yield is directly impacted by sucrose [22,23]. In addition to nutrients, plant hormones also play a determining role in orchestrating plant development [18,24]. CKs are a group of adenine derivatives that facilitate abundant developmental processes in plants, such as vasculature development, maintenance of meristematic cells, differentiation of embryonic cells, and shoot formation [25,26,27,28,29]. Generally, sugars and CKs are individually viewed as major components in many aspects of plant biology. However, the interaction of sugars and CKs has not been systematically investigated [19]. Tissue culture involves the maintenance and development of plant explants placed on artificial media, thus providing a precisely controlled sterile conditions to study the interactions between sugars and hormones regarding plant growth and development.

Here, we artificially constructed different bulblet regeneration systems by adjusting the 6-benzyladenine (BA, an adenine-type CK) concentration in media. The endogenous hormone contents, carbohydrate contents and expression patterns of genes related to sucrose and starch metabolism during in vitro *Ls* bulblet regeneration under different regeneration systems were analyzed. Clonal bulblets of *Ls* were used as explants to avoid the species-specific and genetic background orientated differences. By comparing the established efficient and inefficient regeneration systems, we expected to (1) explore whether and how exogenous CKs affected carbohydrate metabolism and endogenous hormone contents during bulblet regeneration and thus influenced bulb yield and (2) determine the differences in early carbohydrate metabolism between the different regeneration systems. We hope that our results will provide both a theoretical and a practical basis for improving bulblet proliferation efficiency in *Lycoris* species, which would also be useful for exploring the interaction between hormones and carbohydrate metabolism in the production of other bulbous ornamentals.

## 2. Results

### 2.1. Histological Structure of Clonal Bulblets Derived In Vitro

**Figure 1:**
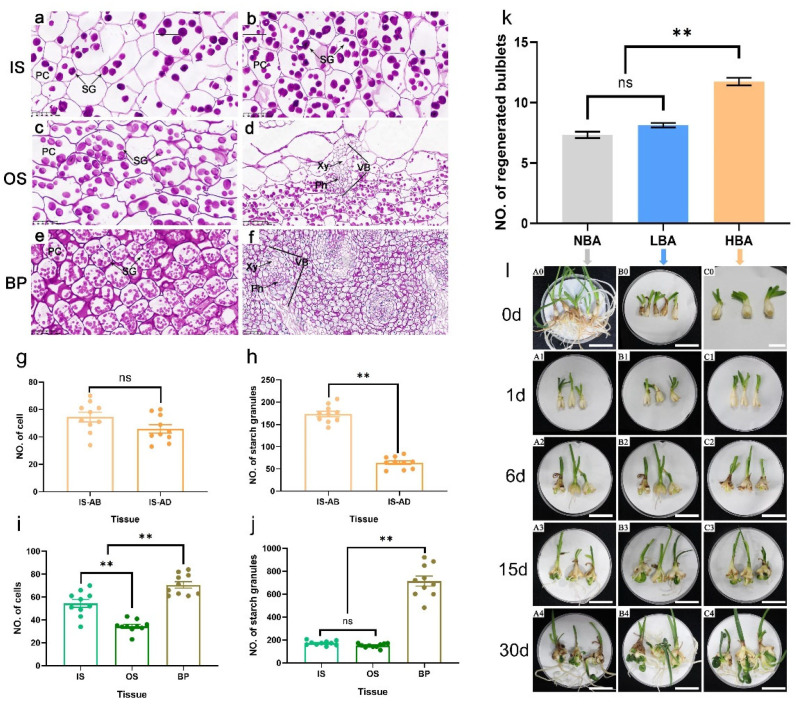
Tissue characteristics of bulblets of *Lycoris sprengeri* and statistical analysis of the regenerated bulblets in response to 6-benzyladenine (BA), treatments. (**a**–**j**): Histological observations and statistical analysis of clonal bulblet of *Lycoris sprengeri*. (**a**) Scale abaxial side, (**b**) Scale adaxial side; Parenchymal cells (**c**) and vascular bundle (**d**) of outer scales; Parenchymal cells (**e**) and vascular bundle (**f**) of basal plate. Number of parenchymal cells (**g**) and starch granules (**h**) in abaxial and abaxial side of scales of clonal bulblet under the same field of view. Number of parenchymal cells (**i**) and starch granules (**j**) in scales and basal plate of clonal bulblet under the same field of view. The solid circles are biological replicate data (*n* = 10). The central values represent the means, and the error bars indicate the SEM of all biological replicates. (**k**) Number of regenerated bulblets in media supplemented with 0, 0.5 and 5.0 mg L^−1^ BA, respectively. Data are represented as the means ± SEM (*n* = 15 biological replicates). (**l**) Morphological observations of bulblet regeneration in three BA treatments from 0 d to 30 d. Bar = 3 cm. NBA, group with no BA treatment, LBA, group with low concentration of BA treatment, HBA, group with high concentration of BA treatment. AB: abaxial, AD: adaxial, IS: inner scales, OS: outer scales, BP: basal plate, SG: starch granules, PC: parenchymal cells, Ph: phloem, Xy: xylem. Asterisks indicate significant differences within each column (Duncan’s multiple range test, ** *p* < 0.01).

Clonal bulblets (diameter = 1 cm) derived from the same probulb were used as plant materials in this study (Figure S1). Significant differences in the shape and number of cells and starch granules between scales and basal plate tissues of bulblets were observed from histological and statistical analysis (Figure 1). Bulblets generally possess four to five layers of scales comprising elliptical-shaped cells, which can be morphologically divided into ISs and OSs (Figure S1). The ISs are much thicker than the OSs, of which the adaxial side is more densely distributed with starch grains than the abaxial side (Figure 1a,b), the same as observed in mature bulbs of *Lycoris* species [4]. Although no significant differences were found in the number of scale cells between the abaxial side and the adaxial side of ISs (Figure 1g), the number of starch granules on the abaxial side was significantly higher than that on the adaxial side under the same field of vision (Figure 1h). The average cell number of ISs was significantly greater than that of OSs in the same unit area (Figure 1i), but the total number of starch grains under the same vision was not significantly different (Figure 1j). The shapes of vascular bundles in scales and basal plates were different. Compared with the compact structure of circular vascular bundles in the basal plate (Figure 1f), the vascular bundles in the scale were loosely distributed with phloem toward the distal axis (Figure 1d). A significantly larger number of approximately circular cells fully filled with smaller starch granules were closely distributed in the basal plate than scale tissues under the same field of view (Figure 1i). Moreover, the number of basal plate-distributed starch granules was significantly higher than that in scales (Figure 1f), indicating the nutrition-transportation role of the basal plate rather than storage.

### 2.2. Effects of BA on Bulblet Regeneration In Vitro of Ls

**Figure 2:**
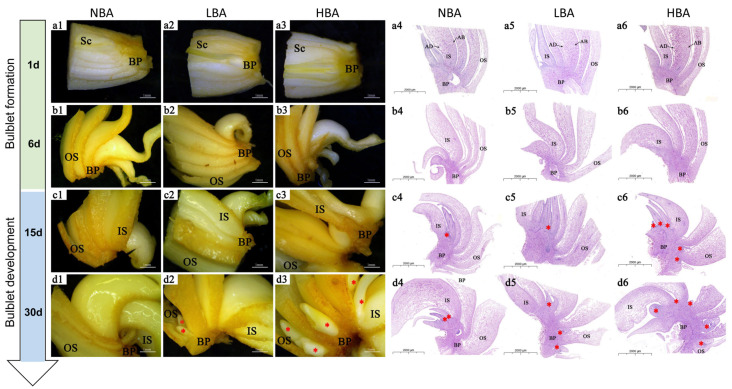
Morphological and histological observations of the in vitro bulblets formation and development process. (**a1**–**d1**,**a2**–**d2**,**a3**–**d3**) represent in vitro bulblets formation and development of NBA, LBA and HBA group under anatomic microscope observation, respectively; (**a4**–**d4**,**a5**–**d5**,**a6**–**d6**) represent in vitro bulblets formation and development of NBA, LBA and HBA group under anatomic microscope observation, respectively. Sc: scale, BP: basal plate, IS: inner scales, OS: outter scales. Red asterisks indicate the newly formed regenerated bulblets.

The application of BA enhanced the bulblet regeneration rate depending on the exogenous BA concentration. The HBA treatment resulted in a significantly higher number of regenerated bulblets (11.73 ± 1.20) than in the LBA treatment (8.13 ± 0.70) and NBA treatment (7.33 ± 1.04), which was used as a comparison (Figure 1k). The whole developmental trends of bulblet regeneration in the two BA treatments were analogous to those of NBA (Figure 1l), albeit with greater number of axillary buds formed in HBA by 15 d after cross-cutting (Figure 2). No significant visible morphological and histological differences were observed by 1 d (Figure 2(a1–a6)). Notably, the scales were significantly thickened during the formation and development of bulblets in vitro (Figure 2(a1–d1,a2–d2,a3–d3)), which was different from the decrease in scale thickness observed during the bulblet formation and development process under aerial culture conditions [4], indicating that scales may play an important role in the accumulation and utilization of exogenous nutrients in vitro.

Histological observations revealed an obvious accumulation of starch granules toward the abaxial side of OS at 6 d in NBA (Figure 2(b4)), which was consistent with our previous observations under aerial conditions [4]; however, no similar distribution trends of starch granules were observed in LBA and HBA by 6 d (Figure 2(b5,b6)). Axillary buds were formed from the connection between the abaxial base of scales and the basal plate in all the treated groups by 15 d (Figure 2(c4–c6)). With the further extension of cell division and nutrient accumulation, the axillary buds gradually grew into bulblets by 30 d (Figure 2(d4–d6)). In our previous study, we found that bulblets of Ls were more distributed in the inner and middle scales, and their outgrowth sequence also showed that the ISs preceded the OSs [4]. Notably, bulblets of NBA and LBA were mainly distributed in the base of IS, whereas the bulblets of HBA occurred at the base of each scale more uniformly, implying that in efficient bulblet regeneration, the incidence of bulblets from OSs is crucial for promoting the bulblet regeneration rate. Accordingly, we divided the in vitro bulblet regeneration process into three main stages: competence stage (0–1 d), bulblet formation stage (1–15 d) and bulblet development stage (15–30 d) (Figure 2).

### 2.3. Effects of BA on Endogenous Hormone Levels during Bulblet Regeneration In Vitro

**Figure 3:**
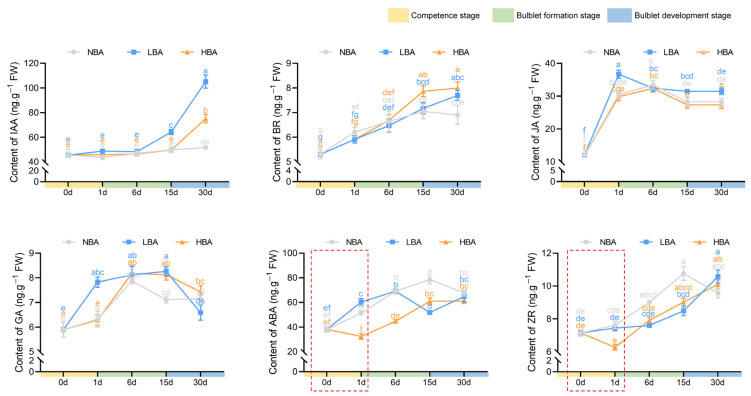
The changes in endogenous hormone contents during the bulblet regeneration process of *Lycoris sprengeri* in vitro. Data are represented as the means ± SEM (*n* = 3 biological replicates). Different letters indicate significant differences at *p* < 0.05 according to Duncan’s multiple range test. The red dashed frames indicate the different changing trend of hormones among groups during the competence stage.

The contents of six major endogenous hormones were measured and compared during bulblet regeneration among the three BA treatments (Figure 3). Indole acetic acid (IAA), the most common auxin, showed the highest content (45.57 ng/g) among the other measured hormones at 0 d in *Ls* bulblets. The content of IAA remained relatively stable during the whole bulblet regeneration process in the NBA group. In contrast, a significant increase in IAA content was observed in the LBA and HBA groups during the bulblet development stage, with a greater increase observed in the LBA group. The brassinosteroid (BR) content in all three treatments showed an upward trend during the bulblet formation stage. Nonetheless, a significantly higher content of BR was observed in the HBA group than in the NBA group during the bulblet development stage. The contents of jasmonic acid (JA) and gibberellic acid 3 (GA3) significantly increased during the competence stage (0–1 d) and then gradually decreased along with bulblet development, and the JA and GA3 contents increased at a faster rate in the LBA group than in the NBA and HBA groups (Figure 3). Intriguingly, changes in the abscisic acid (ABA) and trans-zeatin-riboside (ZR) contents were quite different among the three groups. More specifically, the ABA and ZR contents in the HBA group first rapidly decreased during the competence stage, reaching a minimum at 1 d, and then significantly increased throughout the bulblet formation and development stages (Figure 3). Moreover, we found that the ABA content in the LBA group significantly increased from 0 to 6 d and then decreased from 6 to 15 d, and no decreasing trend was found in ZR content during the competence stage. In comparison, the ABA and ZR contents in the NBA group showed the same trend as that in the LBA group during the competence and bulblet formation stages, whereas an opposite trend was observed during the bulblet development stage (Figure 3).

### 2.4. Effects of BA on Nonstructural Carbohydrate Contents during Bulblet Regeneration In Vitro

**Figure 4:**
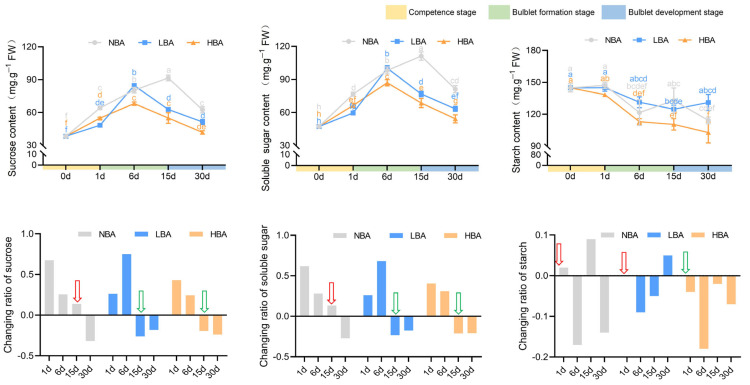
The changes in non-structural carbohydrate contents during the bulblet regeneration process of *Lycoris sprengeri* in vitro. Data are represented as the means ± SEM (*n* = 3 biological replicates). Different letters indicate significant differences at *p* < 0.05 according to Duncan’s multiple range test. The red arrow indicates a positive changing ratio of rate of non-structural carbohydrate contents while the green one indicates a negative changing ratio.

The contents of sucrose and TSS rapidly increased during the competence stage and then decreased with the development of bulblets in all three groups (Figure 4). Notably, application of BA resulted in peaks of sucrose and TSS contents in the HBA and LBA groups by 6 d, which was 9 d earlier than the peaks observed in the NBA group (15 d) (Figure 4). By comparing the changing ratio of sucrose and TSS contents between adjacent sampling times, we found that the application of BA was conducive to the rapid decomposition and utilization of sucrose and TSS. Specifically, the sucrose and TSS contents decreased in the LBA and HBA groups during the late bulblet formation stage (6–15 d), while this decrease in content was not observed in the NBA group until the bulblet development stage (15–30 d). The maximum changing ratios of sucrose content were observed in the NBA (0.67) and LBA (0.75) groups during the competence stage and early bulblet formation stage, respectively. In contrast, the sucrose content in the HBA group changed within a relatively small range (from −0.23 to 0.43). The change in TSS content in the three BA treatment groups was basically the same as that of sucrose, which indicated that sucrose may represent the main component of TSS in *Ls* bulbs (Figure 4).

A decrease in the contents of starch throughout bulblet regeneration was observed in all treatment groups, with the greatest decrease observed in the HBA group (Figure 4). Intriguingly, no positive accumulation of starch was observed in the HBA group throughout the bulblet regeneration process. More specifically, the change ratio of starch between adjacent times was consistently negative during the whole bulblet regeneration process in the HBA group, with the most significant change ratio of −0.183 in the early bulblet formation stage (1–6 d) (Figure 4). Positive change ratios of starch were observed during the competence stage in both the NBA and LBA groups, while negative change ratios were found in the HBA group (Figure 4), indicating that a high exogenously applied content of BA promoted a quicker and more stable decrease in starch.

### 2.5. Correlation Analyses

**Figure 5:**
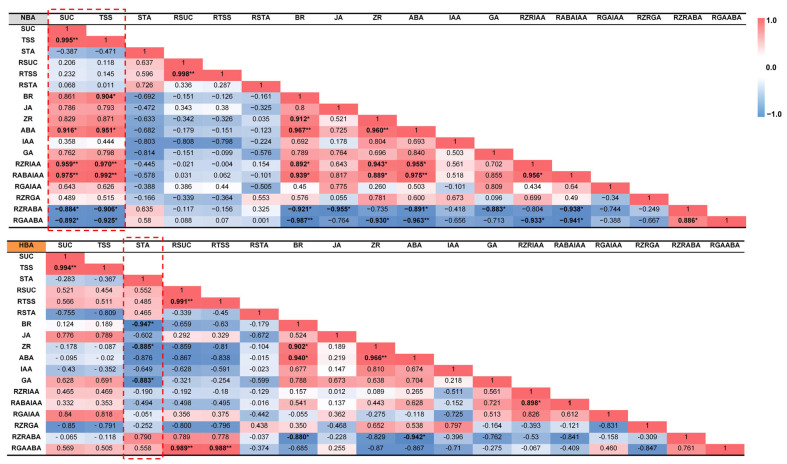
Heatmaps of correlations between endogenous hormone and non-structural carbohydrate indices during in vitro bulblet regeneration of the NBA and HBA groups. * represents significant at *p* < 0.05, ** represents significant at *p* < 0.01. SUC: sucrose, TSS: total soluble sugar, STA: starch, RSUC, RTSS and RSTA indicate the changing ratio of SUC, TSS and STA contents at adjacent time points, respectively. RZRIAA, RABAIAA, RGAIAA, RZRGA, RZRABA, RGAABA indicate the changing ratios between the two hormone contents during the in vitro bulblet regeneration process of *Ls*. For example, RZRIAA represents the ratio of ZR content to IAA content during in vitro bulblet regeneration. Correlation analyses of LBA group is shown in Figure S2, with no significant correlations between endogenous hormone and non-structural carbohydrate indices.

To investigate whether altered exogenous BA treatments affected endogenous hormone homeostasis and nonstructural carbohydrate metabolism, correlations between the hormone indices and contents as well as changing ratios of nonstructural carbohydrates were evaluated in response to each BA treatment. As demonstrated in Figure 5, during the bulblet regeneration process, there was a positive correlation between the contents of BR (0.904*), ABA (0.951*) and TSS in the NBA group. A significant positive correlation was also detected between the sucrose and ABA contents. A significant positive correlation was also detected between the sucrose and ABA contents. When taking the ratio of hormone indices into consideration, the ratio of ZR content to IAA content (RZR/IAA) and RABA/IAA appeared to have positive significant correlations with both sucrose and TSS, whereas negative significant correlations between RZR/ABA and RGA/ABA with sucrose and TSS were observed in the NBA group (Figure 5).

Regarding the HBA group, different correlations were observed (Figure 5). Correlations between BR, ZR, and GA contents and the content of starch were negative during bulblet regeneration in vitro, but the negative correlations between the ratios of hormone indices and sucrose content as well as TSS content became less pronounced with increasing BA concentration in media. In contrast, significant positive correlations were detected between RGA/ABA and R/sucrose (0.989**) and R/TSS (0.988**) (Figure 5). Intriguingly, no significant correlations were observed between the carbohydrate contents and hormone contents in the LBA group, whereas significant positive correlations were detected between RABA/IAA and R/sucrose (0.936**) and R/TSS (0.945**) (Figure 5). It should be noted that the different BA treatments might have changed the interactions between endogenous hormones and carbohydrate contents during bulblet regeneration in vitro.

### 2.6. Expression Patterns of Genes Related to Sucrose and Starch Metabolism during Bulblet Regeneration

**Figure 6:**
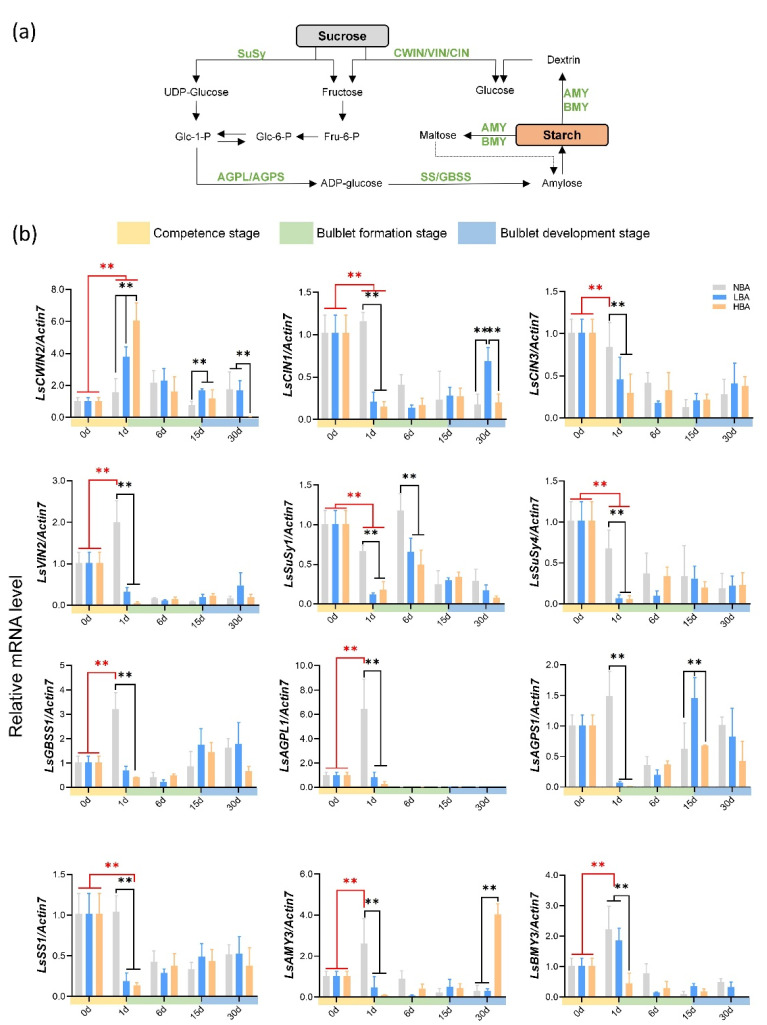
Relative expression of sucrose and starch metabolism-related genes in each group during in vitro bulblet regeneration. Data are represented as the means ± SEM (*n* = 3 biological replicates). SuSy: sucrose synthase, CWIN: cell wall invertase, VIN: vacuolar invertase, CIN: cytoplasmic invertase, AGPL: the large subunit of adenosine 5′-diphosphate glucose pyrophosphorylase (AGP), AGPS: the small subunit AGP, GBSS: granule-bound starch synthase, SS: soluble starch synthase, AMY: alpha-amylase, BMY: beta-amylase. Asterisks indicate significant differences between column (red) or within each column (black) (Duncan’s multiple range test, ** *p* < 0.01).

The mRNA expression levels of sucrose and starch-mobilization-related genes were measured using qRT-PCR (Figure 6). Compared with the bulblet formation and development stages, the competence stage exhibited more significant gene expression differences among the three groups. Intriguingly, two sucrose degradation enzyme-related genes, namely, *cell wall invertase* (*CWIN*) and *SuSy*, exhibited opposite expression patterns during the competence stage (Figure 6). For instance, a 5-fold induction in *LsCWIN2* transcription was observed at 1 d in the HBA group, whereas an 11-fold decrease was observed for *LsSuSy4* during the same period (Figure 6). In addition, the expression of *LsCWIN2* in the HBA group was significantly higher than that in the LBA and NBA groups at 1 d. Notably, *LsCWIN2* was the only differentially expressed CWIN gene in *Ls* during the VP process derived transcriptome data (unpublished), suggesting its critical role. The gene expression changing pattern in *cytoplasmic invertase* (*CIN*) and *vacuolar invertase* (*VIN*) expressed opposite expression patterns during the competence stage in the NBA and BA (LBA and HBA) treatment groups (Figure 6). The expression levels of genes involved in the starch metabolism pathway exhibited significantly higher transcript levels in the NBA group than in the LBA and HBA groups, whereas no significant differences were observed between the LBA and HBA groups during the competence stage (Figure 6).

In the present study, the transcript level of *D-type cyclins* was investigated as an indicator of cell division activity during bulblet regeneration. Changes in the expression patterns of the *LsCycD3-1* and *LsCycD2-1* genes were comparable. In contrast to the rapidly rising expression in the NBA group, a rapid decrease was observed in the LBA and HBA groups during the competence stage. During the subsequent bulblet formation and development stages, the expression of *LsCycD* genes in the HBA group reach a maximum and maintained significantly higher expression levels than in the LBA and NBA groups (Figure S4).

## 3. Discussion

**Figure 7:**
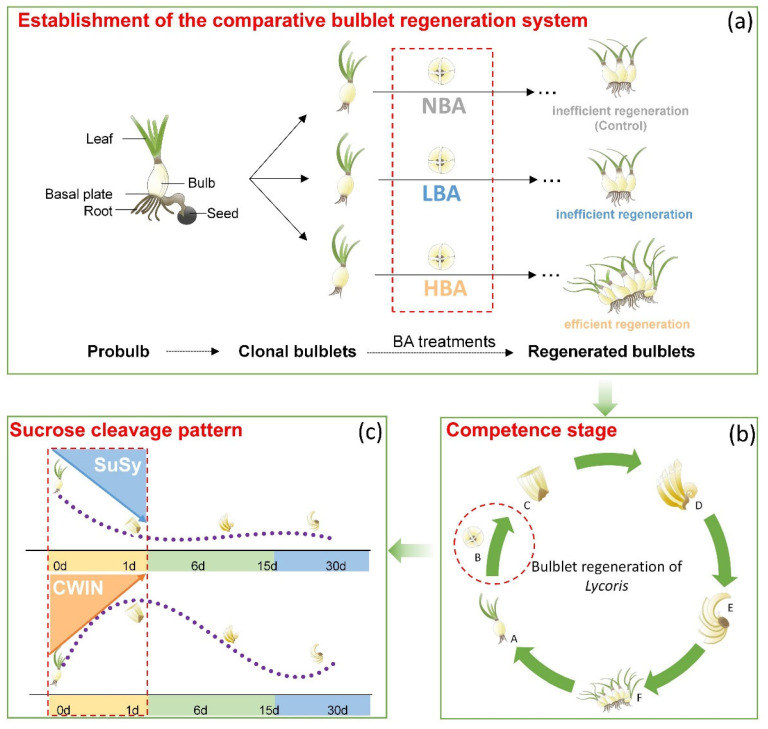
Model of how the early in vitro bulblet regeneration of *Lycoris sprengeri* is regulated from a sucrose cleavage pattern perspective. (**a**) By adjusting the BA concentration in media, we established the efficient and inefficient bulblet regeneration systems. (**b**) Horizontal comparison between groups indicated the pivotal role of the competence stage. (**c**) Bulblet regeneration is likely triggered by sucrose cleavage pattern from sucrose synthase catalyzed to invertase catalyzed at the competence stage, during which a relatively active CWIN-catalyzed sucrose cleavage pattern might lead to an increase in the number of regenerated bulblets of *Ls*, thus influencing bulb yield. CWIN, cell wall invertase; SuSy, sucrose synthase. The average changing patterns of *LsCWIN2* and *LsSuSy4* during in vitro bulblet regeneration of *Ls* are shown in Figure S3.

Tissue culture is a main asexual reproduction method for many flower bulbs and has a significant advantage in promoting regeneration efficiency and shortening the breeding and propagation cycle [8,30]. Axillary shoot formation and outgrowth are critical steps during micropropagation, especially for direct organogenesis via shoot induction, which depends heavily on efficient nutritional allocation and hormone regulation. Although various exogenous factors affecting the in vitro regeneration of *Lycoris* have been continuously optimized, the internal molecular regulatory mechanism of bulblet regeneration remains largely unknown. Here, we presented the first systematic report regarding this biological process in *Lycoris* and made comparisons between the established “efficient” and “inefficient” bulblet regeneration systems (Figure 7). We found that exogenous BA application might affect the incidence of bulblets by manipulating the interaction between carbohydrates and endogenous hormones. Moreover, the rapid sucrose degradation and the dominant sucrose cleavage pattern at the competence stage might play important roles in regulating the incidence of regenerated bulblets.

### 3.1. Establishment of a Comparative In Vitro Bulblet Regeneration System via BA Application

The reproductive rate of *Lycoris* varies among different species [4,13]; however, comparisons between different species could be inevitably affected by their different genotypic backgrounds. Therefore, to exclude the different genotype and cultivar influences on plant regeneration, clonal bulblets of *Ls* derived from the same probulb were used as the explants in this study (Figure S1). Our previous studies confirmed that in a certain concentration range, with increasing BA concentration, the number of regenerated bulblets of *Ls* increased significantly [7]. The current study showed that under different BA conditions, the quantitative characteristics of regenerated bulblets were significantly different (Figure 1); however, their biological processes of bulblet formation and development were highly consistent. Thus, horizontal comparison between groups is feasible. By adjusting the BA concentration in media, we artificially established different bulblet regeneration systems, including efficient and inefficient regeneration systems (Figure 7). Comparisons between the different bulblet regeneration systems with the same genotypic background are expected to reveal the carbohydrate metabolic state and their interaction status with endogenous hormones that are more promotable for bulblet regeneration.

### 3.2. Exogenous BA Mainly Affected the Incidence of Bulblets by Manipulating Hormone Regulation and Nutrient Utilization in the Competence Stage

Sugars and CKs play pivotal roles in plant morphogenesis and development during both the vegetative and reproductive stages of plant life [19]. There are two types of CKs: adenine-type CKs, including kinetin, zeatin and 6-benzylaminopurine (BA), and phenylurea-type CKs, represented by diphenylurea and thidiazuron. BA is the most commonly used exogenous CK in *Lycoris* tissue culture, and it is generally believed that the addition of BA within a certain range is conducive to promoting the occurrence of bulblets [7,31,32]. The positive role of BA in promoting the average proliferation numbers of bulblets has also been reported under ex vitro conditions in *L. radiata* [33]. To further clarify why the addition of BA can significantly promote the incidence of bulblets, we artificially constructed different bulblet incidence systems by adjusting the BA concentration in media. Comparisons among the three groups indicated that the competence stage might be the pivotal period for the interaction between endogenous hormones and carbohydrate metabolism to affect bulblet regeneration in response to different BA concentrations.

Exogenous hormone application is an effective way to promote bulblet development. Xu (2021) examined the effect of different exogenous hormones on bulblet development in *L. radiata* ex vitro and reported that GA significantly inhibited bulblet development, whereas paclobutrazol (PBZ), ABA, and Ethrel had positive effects [33]. In contrast, we found that an initial relatively low level of ABA might be favorable for effective bulblet regeneration in vitro. This difference might result from the different first sampling time points (3 d after cross-cutting by Xu (2021) [33] versus 1 d in this study), indicating that the changes in endogenous hormones are rapid and significant regarding regulation during the competence stage (0–1 d). ZR is the most abundant CK in plants [34], exhibiting cell division and meristematic activities during plant growth and development. ABA was proven to suppress both the content and signaling of CKs in plants [35,36]; however, we did not observe similar phenomena during early in vitro bulblet regeneration of *Lycoris* (Figure 3). Notably, ABA and ZR were the only endogenous hormones that showed opposing changes in contents between the HBA and LBA groups (as well as the NBA group) during the competence stage (Figure 3). In addition, correlation analysis showed that the ZR and ABA contents changed synergistically in the NBA and HBA groups (Figure 5). However, ABA is rarely used in tissue culture, and it remains to be further explored whether the effect of ABA on bulblet proliferation is due to the direct effect on genes involved in the sucrose-to-starch pathway [37,38,39] or the indirect effect caused by the signal interaction with other factors.

Hormones, together with carbon sources and their interactions in the medium, are the key factors affecting plant growth and development in vitro. When the same concentration of carbon source is applied, the correlation analysis of different culture systems indicated that exogenous BA may affect bulblet regeneration by regulating the interaction mechanism between carbohydrates and endogenous hormones (Figure 5). The changes in starch content can reflect the status of sugar metabolism and utilization in bulbs, and starch decomposition usually provides energy for vigorous cell division and differentiation. Much attention has been given to starch metabolism in flower bulbs regarding the bulblet development stage [11,14,40], whereas the role of starch metabolism on quantitative traits during the early formation stage has rarely been studied. At the competence stage, only starch in HBA showed a rapid declining trend (Figure 4), indicating its active starch metabolism and efficient storage energy utilization. Genes related to starch synthesis and metabolism are associated with changes in starch levels [41]. By analyzing key genes involved in starch metabolism, we found that the main reason for the rapid decline in starch content in HBA might be that the expression of the *LsAGPL1*, *LsAGPS1*, *LsGBSS1* and *LsSS1* genes related to starch synthesis was temporarily inhibited during the competence stage. Notably, this phenomenon might result in various contents of glucose-1-P and ADP-glucose, the precursor substances for starch synthesis, among groups and thus affect the selection of sucrose cleavage pathways in bulbs via feedback regulation (Figure 6).

### 3.3. Incidence of Bulblets In Vitro Might Be Associated with the Dominant Sucrose Cleavage Pathway during the Competence Stage

Previous studies indicated that sucrose metabolism holds a central position for bulblet formation and development; for instance, the formation of bulblets in *Lilium* and *Lycoris* was accompanied by the significant upregulation of sucrose catabolism-related enzyme genes and the sharp decline of sucrose content in mother scales [11,17,33]. In addition, sucrose has also been reported to act as signaling molecule instead of an osmotic pressure regulator and carbon source during bulblet induction and expansion in *Lilium sargentiae* [10]. Here, we found that the addition of BA accelerated the decrease in sucrose and TSS, whereas a significant decrease in sucrose and TSS was not observed until the bulblet development stage in the BA deficiency group (Figure 4). In other words, the addition of BA contributes to the rapid degradation and utilization of sucrose. Based on these results, we proposed that efficient sucrose degradation rather than accumulation during competence and early bulblet formation stages might be pivotal for efficient bulblet regeneration (Figure 7).

Sucrose is unloaded from the phloem into sink cells either apoplasmically or symplasmically [22]. For the former, sucrose is hydrolyzed by CWIN into glucose and fructose before being taken up into the cytoplasm (Suc→Glc + Fru). For the latter, sucrose will be symplasmically imported via plasmodesmata (PDs) or taken up by sucrose transporters and then degraded by SuSy (Suc + UDP↔UDP-Glc + Fru) [22,42,43]. In addition to playing a physiological role, both invertase and SuSy can function in sugar sensing and signaling [22,44,45]. Surprisingly, we found that *LsCWIN* and *LsSuSy* were significantly differentially expressed and exhibited nearly opposite expression patterns during the competence stage (Figure 7). *LsCWIN2* expression was significantly upregulated, while *LsSuSys* expression was inhibited during the competence stage (Figure 6). A similar scenario was observed in our previous in vitro study of *Lilium*, in which a successful shoot-to-bulblet transition was associated with a switch from energy production metabolism to storage metabolism and a parallel shift from CWIN-catalyzed to SuSy-catalyzed sucrose cleavage [17]. On this basis, we propose that the dominant sucrose cleavage pattern during the competence stage might influence the bulblet regeneration efficiency of *Lycoris* in vitro. Moreover, we found that the number of regenerated bulblets was consistent with the expression level of *LsCWIN2* at the competence stage; that is, the higher the expression level of *LsCWIN2* was, the more regenerated bulblets we obtained (Figure 7). However, the mechanism by which CWIN promotes bulblet regeneration requires further study.

### 3.4. General Model of the Bulblet Regeneration in Lycoris In Vitro

The formation and development of bulblets in *Lycoris* is similar to that of axillary bud outgrowth in model plants [4,14]. A previous nutritive hypothesis proposed that access to plant nutrients is the major factor regulating axillary bud growth [46,47,48,49,50]. Subsequently, Marson (2014) proposed the initial role of sugar rather than auxin in regulating apical dominance [51]. Here, we found that rapid sucrose degradation at the competence stage was crucial for the formation of bulblets. The relatively active apoplasmic sucrose cleavage pattern hydrolyzed by CWIN is relatively conducive to building an efficient bulblet regeneration system (Figure 7). The signaling role of CWIN has been previously reported; suppressing *CWIN* expression in *Arabidopsis gynoecium* inhibited ovule initiation from the placenta via disruption of the sugar signaling cascade without affecting C nutrient delivery [52]. However, further studies are still needed to obtain a broader spectrum of evidence by monitoring CWIN and SuSy enzyme activities and sugar (e.g., glucose and fructose) levels during in vitro bulblet regeneration. In addition, it is generally accepted that ABA is associated with major plant responses to stress [53], and an inhibitory effect of ABA was observed during bulblet outgrowth in *L. radiata* [34]. However, the effects of changes in early ABA content and its relationship with the sucrose cleavage pattern in *Lycoris* bulbs remain to be explored.

## 4. Materials and Methods

### 4.1. Plant Materials and Sample Collection

A series of clones derived from one probulb of *Ls* were used as explants (Figure S1). Well-developed bulblets were selected and inoculated in rooting medium (MS + 0.5 mg L^−1^ naphthaleneacetic acid + 60 mg L^−1^ sucrose + 3 g L^−1^ phytagel powder (Sigma, St. Louis, MO, USA)) [54] for three months to obtain a sufficient number of 5–9-mm-diameter aseptic explants for bulblet regeneration. Individual bulblets were subsequently transferred to basal MS medium plus 8 g L^−1^ agar and 60 g L^−1^ sucrose with different concentrations of BA: 0 mg L^−1^ (NBA), 0.5 mg L^−1^ (LBA), and 5.0 mg L^−1^ (HBA) based on our previous experiments. A total of 135 bulblets were used for each treatment, with triplicate samples per treatment. Three probulbs were incubated in a glass conical flask (6.5 cm diameter × 10 cm height). The cultures were incubated at 25 ± 2 °C under a 14/10 h light/dark photoperiod with a light intensity of 25 μmol m^−2^ s^−1^ (Philips, Hangzhou, China).

To visualize distinctive bulblet regeneration cytologically, samples containing basal plates and basal scales of bulblets from specific developmental stages of three treatments were examined using the modified periodic acid-Schiff (PAS) method of Mowry (1963) [55] as described by Ren (2017) [4]. Samples for all other measurements were collected from the lower part of bulblets containing basal plates and scales 0 d, 1 d, 6 d, 15 d, and 30 d after cross-cutting. All the samples were frozen in liquid nitrogen for 30 min and then stored at −80 °C.

### 4.2. Endogenous Hormone Determination

Enzyme-linked immunosorbent assay (ELISA) was used to measure the levels of six phytohormones, namely, IAA, ABA, JA, ZR, GA and BR. The ELISA test kits for each plant hormone were purchased from the College of Crop Sciences, China Agricultural University, Beijing, China. The extraction, purification, and determination of the endogenous levels based on ELISA were performed according to Yang (2001) [56], with minor modifications as described by Wu (2017) [9]. Briefly, thoroughly mixed frozen tissues (0.3 g) were homogenized and extracted in 80% (*v*/*v*) methanol with butylated hydroxytoluene (1 mmol L^−1^) for 4 h at 4 °C. The extract was centrifuged at 3500 rpm (4 °C) for 8 min to obtain the supernatant, and the residues were extracted again for 1 h and subsequently centrifuged. The two extracts were combined and the supernatant was purified using a C18 Sep-Pak cartridge (Waters, Milford, MA, USA). The effluent was dried in N_2_ to remove methanol and subsequently dissolved in 1 mL phosphate buffer solution (0.01 mol L^−1^, pH 7.5) for further analysis. The follow-up steps was referring to the ELISA kit instructions. The absorbance was recorded at 490 nm.

### 4.3. Nonstructural Carbohydrate Contents Assay

Thoroughly mixed frozen tissues (0.5 g, fresh weight) were prepared for each sample. Extractions were performed as previously described [30]. Extracted the ground sample with 1.5 mL of 80% chromatographic ethanol for 1 h at 95 °C and then centrifuged at 12,000 rpm for 5 min at 4 °C. The supernatant was collected and extracted with 1.5 mL 80% chromatographic ethanol followed by centrifugation for two times. Filled the supernatant to 50 mL with double distilled water. The concentrations of sucrose and total soluble sugar (TSS) were measured using the modified anthrone method [57]. The remaining pellets were re-extracted for starch determination in accordance with the procedure of McCready (1950) [58]. Added 5 mL 30% perchloric acid to the remaining pellets and then centrifuged (4 °C) for 20 min at 10,000 rpm. The pellets were again re-extracted twice, and the supernatants were combined. Filled the supernatant to 50 mL with double distilled water for the estimation of starch content. The absorbance of the supernatant was read at 620 nm using a Multilabel Reader (Thermo Scientific, Multiskan GO, Waltham, MA, USA).

### 4.4. RNA Extraction and First-Strand cDNA Synthesis

Total RNA extraction was conducted using the EASYspin Plus Plant RNA kit (RN38, Aidlab Bio, Beijing, China) according to the manufacturer’s protocol and treated with RNase-free DNase I (4716728001, 211 Roche, Basel, Switzerland) to remove any remaining genomic DNA. RNA integrity was verified with 1% (*w*/*v*) agarose gel electrophoresis. Quantity and quality were measured with a NanoDrop 2000 Spectrophotometer (Thermo Scientific, Madison, WI, USA). Only RNA samples with absorption ratios of A260/280 = 1.8–2.2 were used in cDNA synthesis. First-strand cDNA was synthesized using a PrimeScript RT reagent Kit with gDNA Eraser (TaKaRa, Dalian, China) following the manufacturer’s protocol. The cDNA was diluted twenty-fold with nuclease-free water for quantitative real-time PCR (qRT-PCR).

### 4.5. Real-Time Reverse Transcription PCR Assays

qRT-PCRs were performed with a TB Green Premix Ex Taq Kit (RR420A, TaKaRa, Tokyo, Japan) in a Bio Rad ConnectTM Optics Module (Bio-Rad, Hercules, CA, USA) following the protocols described by Wu (2021) [17]. Expression data for each target gene in each replicate were normalized to the expression level of the reference gene Actin using the 2^−ΔΔCt^ method [59]. Three biological and technical replicates were performed per treatment. For each treatment of samples, samples at 0 d were selected as the calibrator and assigned a nominal value of 1.0. The values are expressed as the mean of three biological replicates. The standard deviation of the mean is shown as error bars in the figures. The qRT-PCR primers used in this study are given in Table S1.

### 4.6. Statistical Analysis

Statistical analyses of the morphological, physiological and biochemical parameters were performed using one-way analysis of variance (ANOVA) and Duncan’s multiple range test to compare the differences among different sampling stages and treatments. Correlation analysis using the bulblet formation and development parameters of the three treatments was also conducted. All computations were performed using SPSS (IBM, Inc., 22.0, IL, USA). Differences between mean values were subjected to the least significant difference test using a statistically significant level set at *p* < 0.05. Illustrations were generated using GraphPad Prism (GraphPad Software, Inc., 8.0, San Diego, CA, USA) and PowerPoint software (Microsoft Office 365 ProPlus, Redmond, WA, USA).

## Data Availability

Data sharing is not applicable to this article.

## Supplementary Materials

The following are available online at https://www.mdpi.com/article/10.3390/ijms222111890/s1.
