# Supplementary material for: Early Sucrose Degradation and the Dominant Sucrose Cleavage Pattern Influence Lycoris sprengeri Bulblet Regeneration In Vitro
Source: Int J Mol Sci. 2021 Nov 2;22(21):11890. doi: 10.3390/ijms222111890 (PMC8585118; doi:10.3390/ijms222111890)

Figure S1 Probulbs of *Lycoris sprengeri* and BA treatments

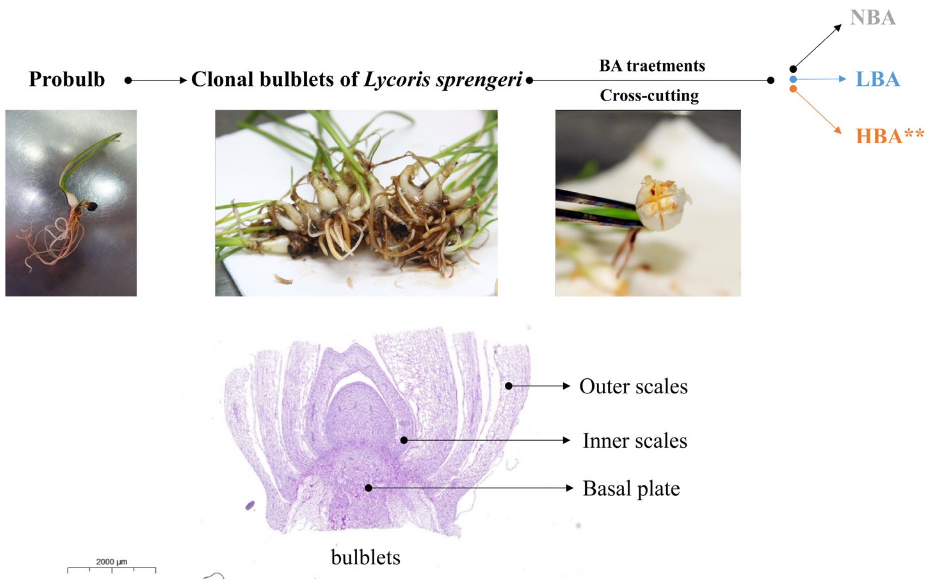

Figure S2 Heatmaps of correlations between endogenous hormone and non-structural carbohydrate indices during *in vitro* bulblet regeneration of LBA group.

LBA	SUC	TSS	STA	RSUC	RTSS	RSTA	BR	JA	ZR	ABA	IAA	GA	RZRIAA	RABAIAA	RGAIAA	RZRGA	RZRABA	RGAABA
SUC	1																	
TSS	0.999**	1																
STA	−0.636	−0.657	1															
RSUC	0.622	0.599	0.202	1														
RTSS	0.611	0.588	0.217	1.000**	1													
RSTA	−0.813	−0.805	0.352	−0.624	−0.621	1												
BR	0.35	0.376	−0.838	−0.367	−0.380	0.144	1											
JA	0.507	0.526	−0.394	0.229	0.237	−0.155	0.552	1										
ZR	−0.021	0	−0.555	−0.519	−0.533	0.54	0.893*	0.312	1									
ABA	0.675	0.681	−0.393	0.499	0.493	−0.187	0.558	0.833	0.4	1								
IAA	−0.109	−0.089	−0.479	−0.551	−0.564	0.614	0.846	0.251	0.995**	0.345	1							
GA	0.73	0.744	−0.496	0.358	0.367	−0.681	0.286	0.78	−0.142	0.542	−0.229	1						
RZRIAA	0.141	0.117	0.526	0.654	0.665	−0.603	−0.866	−0.26	−0.985**	−0.270	−0.987**	0.174	1					
RABAIAA	0.59	0.573	0.217	0.936*	0.945*	−0.695	−0.421	0.358	−0.653	0.432	-0.694	0.569	0.75	1				
RGAIAA	0.4	0.385	0.286	0.724	0.738	−0.751	−0.623	0.136	−0.884*	0.032	−0.918*	0.552	0.914*	0.891*	1			
RZRGA	−0.437	−0.429	−0.109	−0.573	−0.589	0.79	0.48	−0.231	0.811	−0.017	0.858	−0.694	−0.816	−0.800	−0.965**	1		
RZRABA	−0.691	−0.686	0.043	−0.822	−0.828	0.519	−0.019	−0.738	0.226	−0.796	0.282	−0.712	−0.342	−0.874	−0.620	0.573	1	
RGAABA	−0.290	−0.289	0.114	−0.342	−0.332	−0.258	−0.469	−0.493	−0.570	−0.830	−0.564	0.016	0.441	−0.134	0.327	−0.439	0.48	1

Figure S3

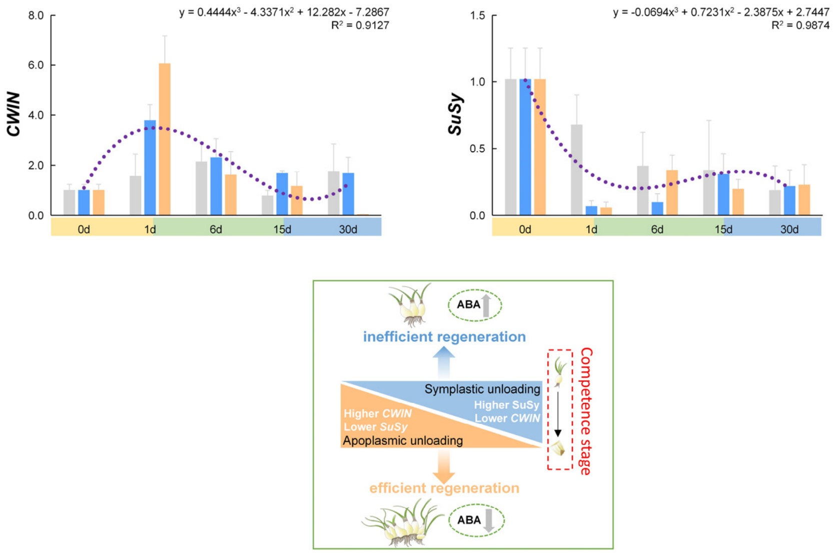

Supplement: Supplementary file 1 [file ijms-22-11890-s001.zip › ijms-1432392-supplementary.pdf]
